# Managing Bardet–Biedl Syndrome—Now and in the Future

**DOI:** 10.3389/fped.2018.00023

**Published:** 2018-02-13

**Authors:** Elizabeth Forsythe, Joanna Kenny, Chiara Bacchelli, Philip L. Beales

**Affiliations:** ^1^Genetics and Genomic Medicine Programme, UCL Great Ormond Street Institute of Child Health, University College London, London, United Kingdom

**Keywords:** Bardet–Biedl syndrome, genetic therapies, pharmacogenomics, genome editing, targeted therapies, drug repurposing

## Abstract

Bardet–Biedl syndrome is a rare autosomal recessive multisystem disorder caused by defects in genes encoding for proteins that localize to the primary cilium/basal body complex. Twenty-one disease-causing genes have been identified to date. It is one of the most well-studied conditions in the family of diseases caused by defective cilia collectively known as ciliopathies. In this review, we provide an update on diagnostic developments, clinical features, and progress in the management of Bardet–Biedl syndrome. Advances in diagnostic technologies including exome and whole genome sequencing are expanding the spectrum of patients who are diagnosed with Bardet–Biedl syndrome and increasing the number of cases with diagnostic uncertainty. As a result of the diagnostic developments, a small number of patients with only one or two clinical features of Bardet–Biedl syndrome are being diagnosed. Our understanding of the syndrome-associated renal disease has evolved and is reviewed here. Novel interventions are developing at a rapid pace and are explored in this review including genetic therapeutics such as gene therapy, exon skipping therapy, nonsense suppression therapy, and gene editing. Other non-genetic therapies such as gene repurposing, targeted therapies, and non-pharmacological interventions are also discussed.

## Introduction

Bardet–Biedl syndrome (BBS), sometimes known as Laurence–Moon–Bardet-Biedl syndrome, is a rare autosomal recessive ciliopathy characterized by rod-cone dystrophy, learning difficulties, polydactyly, obesity, genital malformations, and renal abnormalities.

In the 1880s, a family with retinitis pigmentosa, obesity, and intellectual impairment was described by doctors Laurence and Moon. The affected family members later went on to develop a spastic paraparesis. In 1920 and 1922, respectively, doctors Bardet and Biedl independently described two families with obesity, retinitis pigmentosa, and polydactyly. From 1925, the syndrome was known as Laurence–Moon–Bardet–Biedl syndrome, but there was disagreement as to whether they were the same entity. Later, it was considered as two entities, Laurence–Moon and Bardet–Biedl syndromes, but mutations in known BBS genes have been seen in families with both syndromes (1, 2). Today, it is most usually known as BBS.

It is a pleiotropic disorder and has a prevalence of around 1:100,000 in North America and Europe, but it is significantly more common in certain isolated communities including Newfoundland (1:18,000) (2) and Kuwaiti Bedouins (1: 13,500) (3, 4). In the last 2 decades, 21 BBS genes (BBS1–BBS21) (5–7) have been identified, mutations in which account for 80% of cases with a clinical diagnosis of BBS (1). Table 1 outlines the 21 BBS genes.

**Table 1:** Known genes causing BBS.

BBS type	Gene name
BBS1	*BBS1*
BBS2	*BBS2*
BBS3	*ARL6*
BBS4	*BBS4*
BBS5	*BBS5*
BBS6	*MKKS*
BBS7	*BBS7*
BBS8	*TTC8*
BBS9	*BBS9*
BBS10	*BBS10*
BBS11	*TRIM32*
BBS12	*BBS12*
BBS13	*MKS1*
BBS14	*CEP290*
BBS15	*WDPCP*
BBS16	*SDCCA8*
BBS17	*LZTFL1*
BBS18	*BBIP1*
BBS19	*IFT27*
BBS20	*IFT172*
BBS21	*C8orf37*

Mutations in *BBS1* and *BBS10* account for the majority of genotypes (~51 and ~20%, respectively) in Northern Europe and North America (4).

Bardet–Biedl syndrome proteins localize to the primary cilium/basal body complex, a ubiquitously expressed highly evolutionarily conserved organelle functioning primarily for cell-to-cell signaling. The genes that cause BBS can also cause other ciliopathies, with the classic example being *CEP290*, which can cause Joubert syndrome, Leber congenital amaurosis, Meckel syndrome, and Senior-Loken syndrome in addition to BBS (8).

## Diagnosing BBS

**Figure 1:**
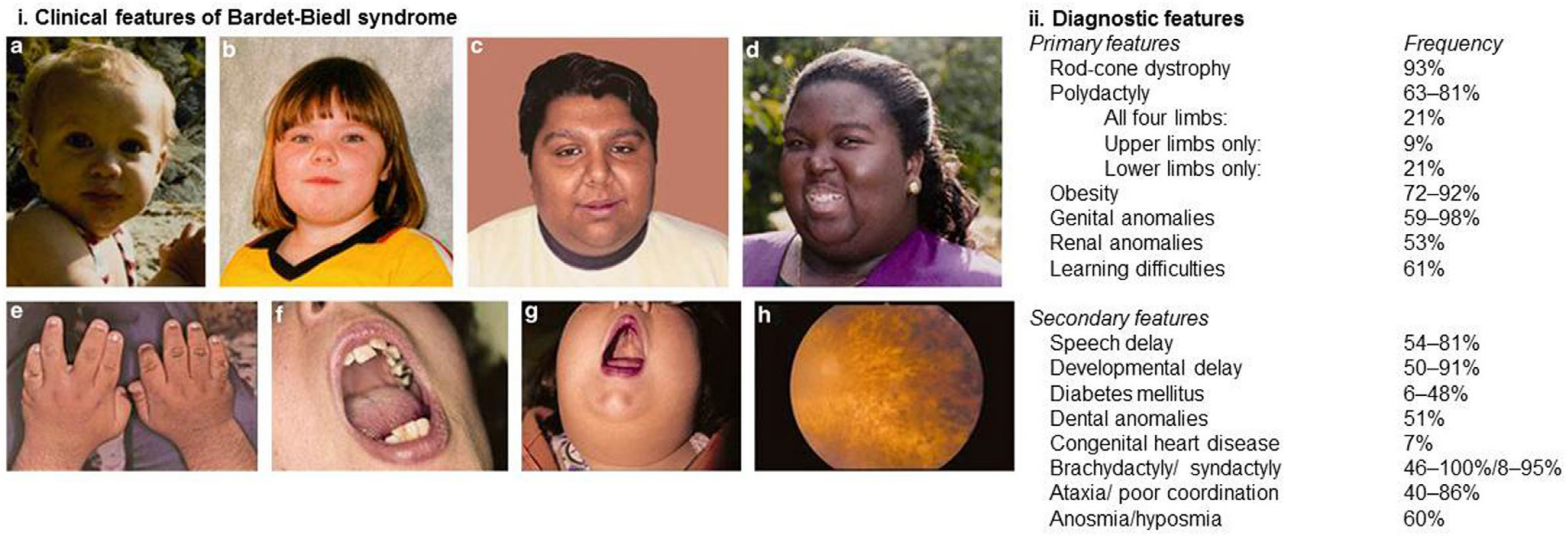
Clinical and diagnostic features of Bardet–Biedl syndrome. (i) Clinical features associated with Bardet–Biedl syndrome. **(A–D)** Typical facial features are often subtle and not always present. Typical facial features include malar hypoplasia, a depressed nasal bridge, deep set eyes, and retrognathia. **(E)** Brachydactyly. **(F)** Dental crowding. **(G)** High palate. **(H)** Rod-cone dystrophy. (ii) Diagnostic features of Bardet–Biedl syndrome. At least four major features or three major and two minor features are required to make a clinical diagnosis. Informed consent was obtained and republished with permission (4).

Bardet–Biedl syndrome is a pleiotropic disorder and diagnosis is based on the presence of at least four major features or three major features and at least two minor features in accordance with the diagnostic criteria published by Beales et al. (9). Figure 1 demonstrates the clinical features associated with BBS and highlights the relative frequencies at which these features are observed.

Molecular confirmation of BBS has evolved over the last decade from targeted sequencing of common genetic variants, including the common *BBS1* p.M390R, *BBS2* p.Y24X, *BBS2* p.R275X, and *BBS10* c.91fsX5 mutations to next-generation sequencing gene panels containing all known BBS genes. The frequency at which molecular confirmation is achieved has increased accordingly from approximately 40–80% (1).

The age at which patients are diagnosed is extremely variable and is driven by the age of onset of symptomatic rod-cone dystrophy. While this may manifest in infancy, it is more usually seen between the ages of 5 and 10 years of age and typically presents with night blindness (9). Isolated polydactyly at birth or obesity, generally seen from infancy, do not usually prompt referral. Siblings of affected children are generally diagnosed earlier. Antenatal diagnosis is extremely rare in the absence of a family history, but BBS may be suspected from the identification of echogenic kidneys and polydactyly on ultrasound scanning. Children presenting with renal anomalies or renal failure may be diagnosed earlier than those without, but there are insufficient data to confirm this. A subset of individuals present with isolated rod-cone dystrophy with notable absence of other BBS-related features and are often diagnosed in adulthood. These individuals are now being picked up because of the introduction of panel-based genetic testing and major diagnostic studies such as the UK 100,000 genomes project (10) and the Deciphering Developmental Disorders (exome) study (11). They were previously overlooked as there are many causes of rod-cone dystrophy, and it was not understood that BBS genes could cause this feature in isolation.

Currently, diagnostic gene panels are the diagnostic tool of choice. The use of whole exome sequencing (WES) and whole genome sequencing (WGS) may increase coverage, aid in the discovery of novel genes, and allow for the identification of non-coding variants. However, along with increased expense, disadvantage of the more advanced diagnostic sequencing techniques is the identification of pathogenic variants in non-BBS genes and of variants of unknown significance (VUS) in BBS genes (12). This can result in a diagnostic conundrum in particular where patients manifest only one or two non-specific major diagnostic criteria such as obesity and/or learning difficulties. The use of WES and WGS requires careful consenting of patients and having a plan in place to deal with VUS and unlooked for results.

Variable expressivity is the hallmark of BBS (8); patients with the same genotype and even siblings frequently manifest symptoms differently. As a result, although genotype–phenotype correlations exist on a population basis, it is not possible to make individual predictions about symptomatic manifestations (13). As a group, patients with mutations in *BBS1* are usually less severely affected than patients with mutations in other BBS genes. On average, they develop visual deterioration later in life (14), are less likely to develop renal disease (13), and more likely to have a better endocrine biochemical profile (15) with a lower prevalence of metabolic syndrome (16, 17). It is not possible to delineate if this is a consequence of the common missense mutation *BBS1* p.M390R identified in 80% of Northern European patients (4) or if the milder phenotype is representative of an overall less severe phenotype associated with *BBS1* mutations (17).

Suggestion that BBS would be a candidate for triallelic inheritance, whereby a third mutation is required to either manifest the condition or adding mutational load, has gathered limited evidence (18–20). In practice, the phenotypic variability observed in patients with the same genotype and within families is likely to reflect a complex interplay between multiple genetic factors and environmental influences.

In the future, it may be possible to identify phenotypic modifiers and further elucidate the cause for variability through analysis of the “Omics” (genomics, epigenomics, transcriptomics, proteomics, and metabolomics) whereby the complex interplay of genes, transcription, protein expression, and metabolism is considered as part of the phenotypic analysis (21, 22).

## Renal Disease in BBS

Bardet–Biedl syndrome has classically been associated with polycystic kidney disease, a typical feature of ciliopathies with renal manifestations (13, 16, 23–29). The prevalence of renal disease in BBS has been estimated at 53–82% (13, 16, 25). A recent study of 350 patients from the United Kingdom estimated that 50% of patients will develop functional renal disease and demonstrated that cystic or dysplastic disease only accounts for 30% of patients with renal disease, where the remainder have hydronephrosis, scarred or atrophic kidneys, loss of corticomedullary function, or developmental abnormalities (13). Around 8% of patients go on to develop end-stage renal disease requiring dialysis or transplantation (13). The majority of patients who develop end-stage renal disease do so in early childhood (before the age of 5), and in most cases, deterioration is rapid with frequent requirement for dialysis within the first year of life (13). Some patients develop sudden renal failure in adulthood for unknown reasons, and a further group of patients develop end-stage kidney disease as a result of comorbidities including type 2 diabetes and hypertension (13). The prevalence of these comorbidities is thought to be higher in BBS patients than the normal population and in 1 study of 69 patients were found in 22 and 35%, respectively (17). The risk of type 2 diabetes relates to obesity and is treated using standard protocols. Many patients with structural renal abnormalities do not go on to develop functional renal disease (17).

The molecular mechanistic pathways leading to renal disease in BBS remain unelucidated (29). It has been suggested that aberrant mTOR signaling may contribute to the development of cystic kidney disease (28). Another theory proposes that ciliary dysfunction leads to aberrant non-canonical Wnt signaling and planar cell polarity, which may contribute to the development of cysts (30). Molecular evidence supporting these theories remains limited. Furthermore, they do not explain why only some patients develop renal dysplasia or indeed the heterogeneous types of renal disease developed by patients.

## Current Management of BBS

Bardet–Biedl syndrome is currently treated symptomatically focusing in particular on aggressive management of diabetes, hypertension, and metabolic syndrome to minimize the secondary impact that these conditions have on vulnerable organ systems already affected by BBS, in particular the eyes and kidneys (17). Weight management is a continual struggle for the majority of patients (31). Some elect to have bariatric surgery (32) while others take antiobesity medication, but for the majority of patients, dietetic input provides the safest and most effective weight loss strategy (33).

In the UK, patients who attend the national BBS clinics are invited to attend a multidisciplinary clinic for annual review by a geneticist, ophthalmologist, nephrologist, endocrinologist, psychologist, dietitian, speech and language therapist, nurse, and a patient support group representative. This provides a platform for regular review and individualized risk assessment in particular with reference to renal and endocrine deterioration. All patients are genotyped using a service-developed diagnostic gene panel. The clinic also provides an opportunity for research into the natural progression of BBS, and it is expected that patients will eventually be stratified according to genotype and their need for clinical follow-up.

## Future Therapies for BBS

**Figure 2:**
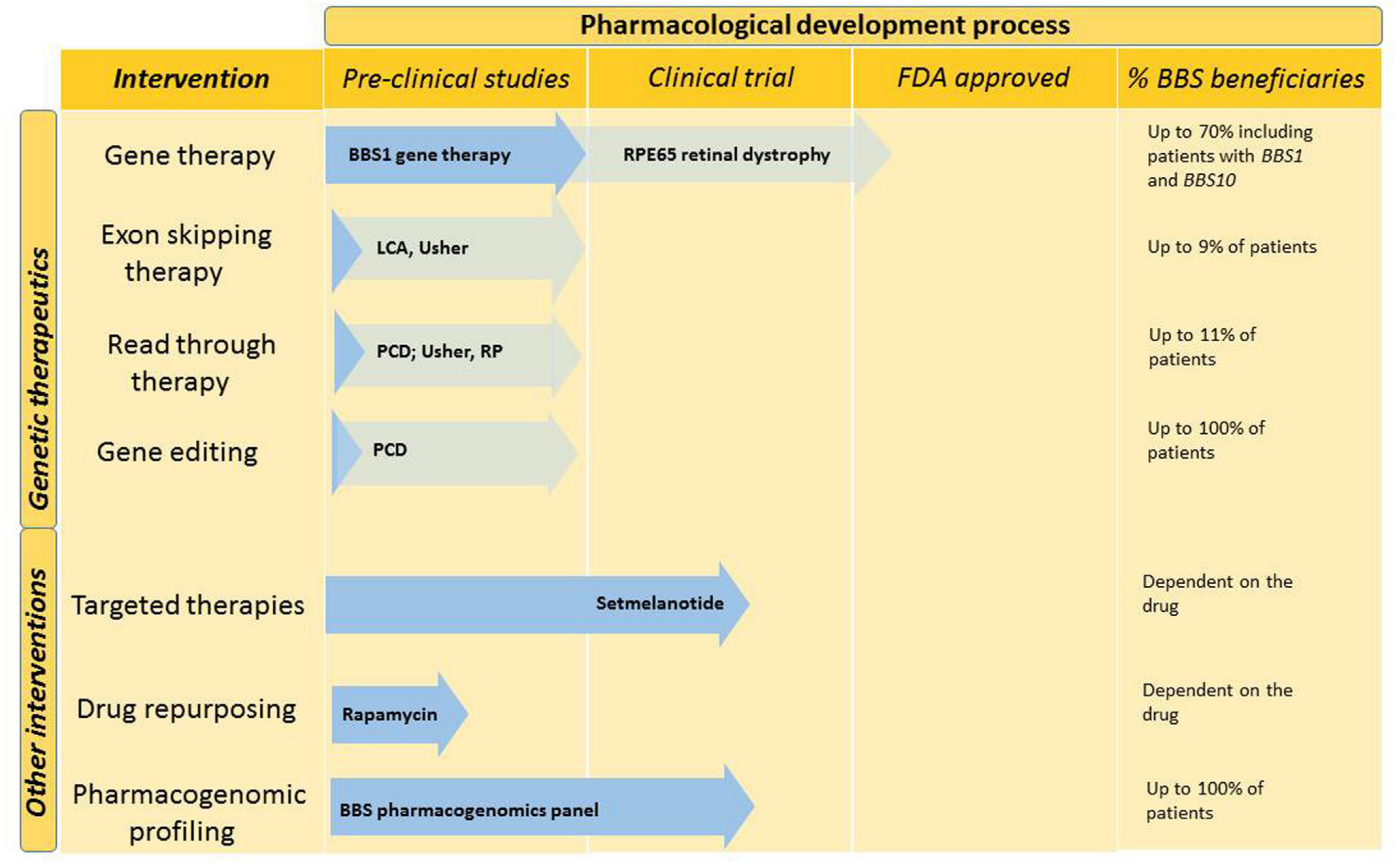
Future interventions and stage in the pharmacological development process. Genetic therapies and other pharmacological interventions are under development for BBS. Dark blue arrows demonstrate the stage to which BBS-specific interventions have been developed. Other ciliopathy relevant developments are indicated in light blue. The last column indicates the percentage of BBS patients who could benefit from this type of intervention. BBS, Bardet–Biedl syndrome; LCA, leber congenital amaurosis; PCD, primary ciliary dyskinesia; RP, retinitis pigmentosa; US, Usher syndrome.

The last decade has seen significant advances in the development of therapeutic modalities, which could potentially be applicable to patients with BBS and related ciliopathies. However, the large number of disease-causing genes and private mutations, which are those seen in only a single family, present a unique challenge in developing genetic therapies for BBS (34). Figure 2 outlines potential future therapies, which are likely to benefit patients with BBS in the future.

### Genetic Therapies

A particular focus for therapeutic intervention in the ciliopathies has been the development of therapy for rod-cone dystrophy (34–36). The eye offers an attractive organ for therapeutic intervention in BBS due to the ease of access, the presence of a control (other) eye, the small amount of tissue that needs to be infiltrated, and a window of opportunity to develop treatment as patients typically do not develop symptoms before mid to late childhood (37).

Novel disease models are being generated with the potential to develop *in vitro* organ systems for the assessment of new therapies. A promising development is the generation of induced pluripotent stem cells (38–43). These cells are generated when adult cells are reprogrammed and subsequently differentiated into another cell type through the addition of growth factors (41). A number of cell types have been used for reprogramming including dermal fibroblasts (44), renal epithelial cells (45), keratinocytes (46), and peripheral blood cells (47).

Urine-derived renal epithelial cells have been used to model ciliopathies, such as Joubert syndrome to assess the effect of potential therapeutics (48, 49) and BBS to derive mechanistic insights into disease pathogenesis (unpublished data). This is a particularly attractive model for many ciliopathies including BBS as it is non-invasive and offers an organ-specific relevant disease model.

To our knowledge, currently, there are no efforts in progress to develop genetic therapies targeted to the renal manifestations of BBS. This is likely to be a function of a number of issues including that the onset of renal manifestations is often antenatal precluding a therapeutic window of opportunity. Furthermore, the natural history of renal disease in BBS is not well understood, and the cause of renal disease can be both primary (e.g., cystic renal disease) and secondary to metabolic syndrome, hypertension, or diabetes all of which occur more frequently in BBS than the general population (13). In addition, the kidney is more difficult to target with genetic therapies but may prove to be a more amenable target for pharmacological therapies.

#### Gene Therapy

Traditional gene replacement therapy has achieved significant success in the treatment of ciliopathy-related eye diseases including Usher syndrome and Leber congenital amaurosis in recent years (50–52). The premise involves the generation of viral or non-viral vectors carrying a wild-type gene of choice with the aim of integrating the gene into the host genome. The highest chance of success is achieved in diseases where only a small amount of healthy gene expression is required to generate a phenotypic effect (53).

A major challenge in developing genetic therapies for BBS is the generation of a long lasting therapy. A successful example of this is the retinal gene therapy (Luxturna), which has been successfully developed for RPE65-associated Leber congenital amaurosis. It is likely to obtain approval from the Food and Drug Administration (FDA) in 2018 as the first gene therapy for ocular disease. Extensive optimization was required before RPE65 gene therapy could be launched for FDA approval due to concerns about the sustainability and long-term maintenance of visual function (36, 37, 54).

Work on retinal gene replacement therapy for BBS is ongoing in animal models, and recent efforts were published demonstrating encouraging results in knock-in mouse models with the most common genotype in humans *BBS1* p.M390R (55). Viral AAV vectors were generated containing the wild-type *Bbs1* construct and injected subretinally rescuing BBSome formation and rhodopsin localization and showing trends toward improved electroretinogram function in mice (55).

Challenges of developing safe and effective gene therapy include ensuring that the gene expression is proportionate to the requirements of the host organism avoiding overexpression and thus cell toxicity (36, 51, 55), identifying an appropriate time to administer therapy (36) (in the case of rod-cone dystrophy ideally before photoreceptor death), avoiding generation of an immune response, and developing safe and effective vectors (53).

#### Readthrough Therapy

Readthrough therapy exploits the natural inconsistency of the genetic proofreading mechanism in the process of RNA translation into protein (56). Nonsense mutations account for an estimated 11% of the total mutational load in BBS (own unpublished data) and lead to premature termination of protein synthesis and subsequent degradation via nonsense mediated decay (57). Readthrough therapy acts by destabilizing the translational ribosome’s response to a nonsense mutation, hence allowing the insertion of a near cognate amino-acyl-tRNA. This allows translation to continue and a full-length protein to be produced (58). The effect mimics that of a missense mutation in the same locus, which may result in a less severe clinical phenotype. Readthrough therapy has been applied to a number of different conditions and been tested in clinical trials for both cystic fibrosis (59–61) and Duchenne muscular dystrophy (62, 63). The effect of readthrough therapy has been assessed on some ciliopathies at the preclinical stage including primary ciliary dyskinesia (64), Usher syndrome (65), and retinitis pigmentosa (RP2) (66).

#### Exon Skipping Therapy

Exon skipping therapy operates at the level of RNA transcription allowing the transcriptional machinery to “skip” exons containing undesirable genetic sequences (67, 68). Antisense oligonucleotides are designed to target an exon/intron of interest. This allows for a novel splicing product that may retain much of its wild-type function depending on the quantity and importance of functional motifs coded for by the exon. This form of genetic therapy is often used to target mutations, which disrupt the genetic reading frame and may otherwise result in a complete lack of functional protein. Successful application of exon skipping therapy has been developed to the level of clinical trials for Duchenne muscular dystrophy and spinal muscular atrophy (69, 70). Exon skipping therapy for ciliopathies has been developed to the stage of preclinical trials for Leber Congenital Amaurosis and Usher syndrome (67, 71). In BBS, this form of therapy could benefit up to 9% of patients. The primary challenge is that many mutations suitable for exon skipping therapy in BBS are private and thus require truly individualized therapy. The common frameshift mutation in BBS (*BBS10* c.91fsX5) is not a suitable candidate as the gene contains only two exons.

#### Genome Editing

Genome engineering offers an attractive future prospect in the management of genetic diseases, allowing DNA to be deleted, replaced, or corrected (72). Targeted endonucleases create double-stranded breaks at specific points in the genome allowing for DNA repair, which can restore the wild-type genotype (43). Promising results have been achieved in cells from patients with the motile ciliopathy primary ciliary dyskinesia where ciliation was restored on replacement of the wild-type *DNAH11* sequence (73). Preclinical work and clinical trials for other diseases using genome editing techniques are progressing with significant advances in animal models of epidermolysis bullosa (74). Initial reports of successful gene editing in humans include gene editing on embryos at risk for hypertrophic cardiomyopathy caused by *MYBPC3* mutation (75) and treatment-resistant leukemia (75, 76). The potential applications are promising, but this technique requires significant refinement in specificity, efficacy, and safety before this can be applied in a clinical setting. Off target effects, whereby genome engineering may erroneously occur on an unintended gene causing DNA damage are a major challenge in bringing this technique forward (72).

While genome editing can be used to target specific organ systems, it can also be applied *ex vivo* to correct or insert mutations to directly comparable cell types which apart from the mutation of interest are genetically identical, thus eliminating genetic background noise and providing an ideal model system (77).

### Non-Genetic Personalized Therapies

#### Targeted Therapies

A novel development in the therapeutic landscape for BBS is the application of targeted drug therapies. An example of this is the ongoing work on the melanocortin receptor agonists as potential therapeutic intervention against obesity in BBS (78). Our understanding of how aberrant BBS proteins cause signal disruption is still evolving; however, there is emerging evidence suggesting that BBS results in defects in the hypothalamic leptin–melanocortin axis (79). This in turn causes leptin resistance culminating in obesity (78).

Seo et al. demonstrated that intravenous administration of a melanocortin receptor agonist decreased both body weight and food intake in wild-type and *Bbs* knockout mice (78). A phase 2/3 clinical trial is in process assessing the effect of novel melanocortin receptor agonist setmelanotide on obesity in BBS and other forms of syndromic obesity (80).

#### Drug Repurposing

Future therapies may include repurposing of drugs, which are already FDA approved. Early work on *Bbs* zebrafish indicated that rapamycin may be a candidate for rectifying the renal cystic phenotype (81). Further work on higher animals has not been published, but drug repurposing offers an attractive and economical option for management in a clinical setting since the cost and failure rate of developing novel therapeutics remain high.

## Non-Pharmacological Future Interventions

A major challenge in any clinical service is harnessing the advances in technology and managing increasing clinical pressures. To this end, the UK National BBS clinic is implementing several new strategies to meet the increasing need for transparency and access to clinical services.

There is an increasing move to offer patients easy access to their medical notes and to data sharing within the UK National Health Service to optimize patient management (82). These issues are being addressed in the UK National BBS clinic through the development of a cloud-based medical notes system, which is accessible to all clinicians in the service. In the future, patients will be able to access their personal medical records through smart phones or other devices, so that medical information can be accessed wherever they are.

Telemedicine and virtual clinics, whereby patient consultations can take place *via* a screen in the patient’s own home, are an attractive alternative for BBS patients who are stable and in particular for those with visual impairment where travel can present a considerable obstacle to attending hospital appointments. It also offers an advantage to clinicians where hospital resources and clinic space are at a premium.

## Pharmacogenomics

Pharmacogenomic profiling, whereby the effect of the genome on drug response is determined, could offer significant advantages to those patients with complex medical needs who are subject to polypharmacy (83) including BBS patients. Although several private companies offer pharmacogenomics gene panels, this is not currently available in the UK National Health Service, and the evidence base is unclear for many of these private initiatives. However, there is a growing evidence base supporting pharmacogenomic profiling, and this is likely to become available in the National Health Service in the coming years.

## Conclusion

Bardet–Biedl syndrome provides a robust model disease for future opportunities in genetic and non-genetic therapeutic management of rare diseases. A significant advantage in the United Kingdom is the presence of the national BBS clinics, which offer a centralized hub for clinical and scientific expertise. The multiorgan effects and wide range of genes and mutation types mean that a number of different genetic therapeutic modalities must be considered, as well as non-genetic pharmacological interventions and non-pharmacological approaches to optimize management of this rare disease in the future.

## Author Contributions

EF, JK, CB, and PB made substantial contributions to the writing, drafting, and revision of this manuscript. All authors approved the final published version of the manuscript.

## Conflict of Interest Statement

The authors declare that the research was conducted in the absence of any commercial or financial relationships that could be construed as a potential conflict of interest.
